# A Case of Stercoral Perforation Detected on CT Requiring Proctocolectomy in a Heroin-Dependent Patient

**DOI:** 10.1155/2016/2893925

**Published:** 2016-10-18

**Authors:** William H. Seligman, Fahreyar Alam, Andy Planner, Roderick J. Alexander

**Affiliations:** ^1^Department of Surgery, The Great Western Hospital, Swindon, UK; ^2^Department of Radiology, The Great Western Hospital, Swindon, UK

## Abstract

Stercoral perforation of the colon is rare but carries with it significant morbidity and mortality. Stercoral perforation usually occurs in elderly, immobile patients with chronic constipation. In this manuscript, we report the case of stercoral perforation in a patient due to chronic heroin dependence. We report the case of a 56-year-old male patient with stercoral perforation, diagnosed by computed tomography, secondary to heroin dependence, requiring proctocolectomy and an end ileostomy. There are very few reports in the literature describing cases of stercoral perforation and questions have been asked about the importance of preoperative cross-sectional imaging. In our case, the diagnosis of stercoral perforation was made only on CT. Although this is not the first such case to be reported, it is significant as preoperative CT imaging was influential not only in determining the aetiology of the abdominal distension seen on the plain film, but also in detecting the pneumoperitoneum which was not evident clinically or on plain radiographs.

## 1. Introduction

Perforation of the colon is most commonly seen in patients with diverticular disease, malignancy, or inflammatory bowel disease. A rare cause of colonic perforation is rupture of the wall of the colon as a result of pressure from within, stercoral perforation. Stercoral perforation is most commonly seen in immobile patients with chronic constipation [1]. Very few cases of stercoral perforation have been reported in the literature [2]. Stercoral perforation is associated with significant mortality (35%) and thus early diagnosis is essential [3]. In this manuscript, we report the case of stercoral perforation as a consequence of constipation from chronic heroin use. This is one of very few such reports in the literature [4, 5]. Unlike previous reports, our patient did not present with clinical signs of gross peritonitis and plain radiographs did not demonstrate pneumoperitoneum. Nevertheless, the patient had a significant stercoral perforation (evident on CT) requiring proctocolectomy and an end ileostomy.

## 2. Case Presentation

Written informed consent was obtained from the patient for publication of this case report and accompanying images. A copy of the written consent is available for review on request.

A 56-year-old intravenous drug user presented to the Surgical Assessment Unit with a several months' history of gross abdominal distension, constipation, and pain. The patient had not opened his bowels for at least three months prior to admission. The patient had had sudden onset of acute abdominal pain in addition to his other symptoms several days prior to admission. In order to cope with the pain, the patient had started to inject heroin into his abdominal wall.

On examination, the patient was in visible pain. Observations were as follows: temperature 38°C, blood pressure 140/90 mmHg, pulse 95 beats per minute, oxygen saturations 97% on air, and respiratory rate 24 breaths per minute. Examination of the abdomen revealed significant distension and diffuse tenderness although there was no obvious peritonism. On digital rectal examination, there was faecal soiling and significant faecal loading.

**Figure 1:**
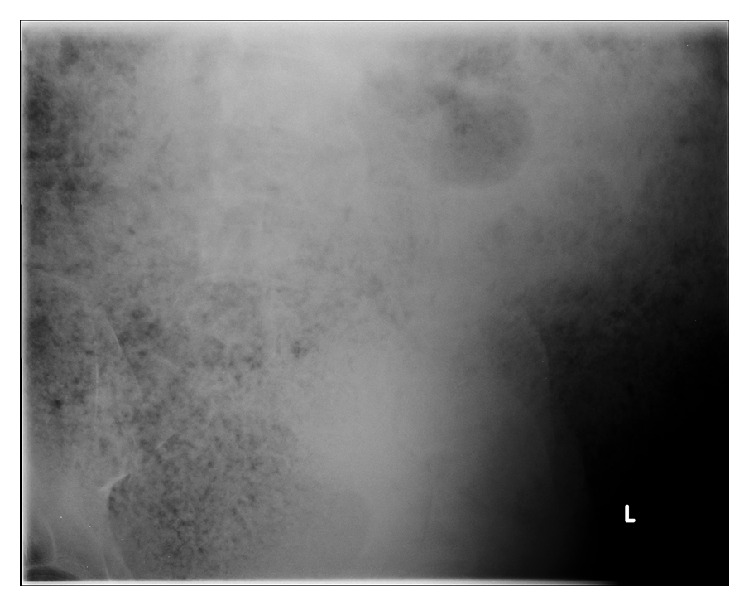
Plain preoperative abdominal radiograph.

**Figure 2:**
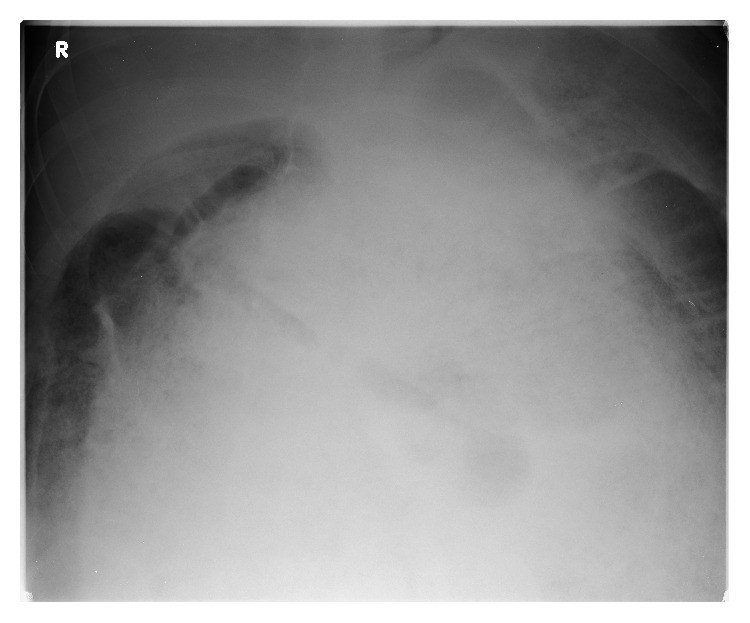
Plain preoperative erect chest radiograph.

**Figure 3:**
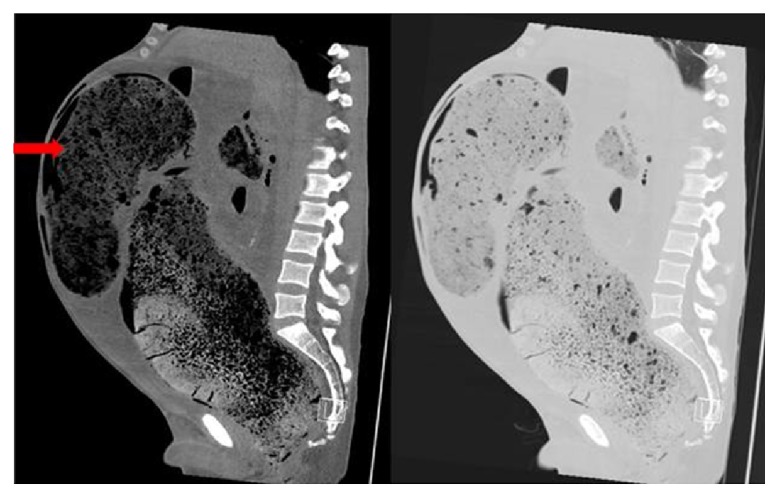
Sagittal MPR CT images with soft tissue windows on the left and lung windows on the right. There is gross chronic colonic distension with faecal loading down to the low rectum. The arrow shows faeces in a capacious sigmoid colon. No free gas on the film.

**Figure 4 fig4:**
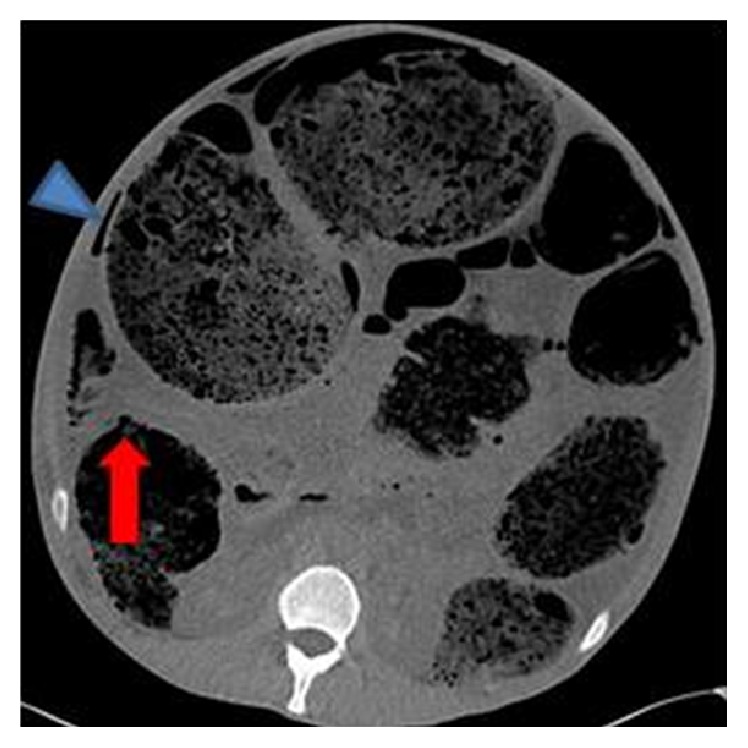
Axial CT images on soft tissue windows showing the anterior ascending colon perforation site (arrow). The ascending colon has collapsed a little relative to the distal bowel. No definite intramural gas in the wall. There is right-side pneumoperitoneum (arrowhead).

**Figure 5 fig5:**
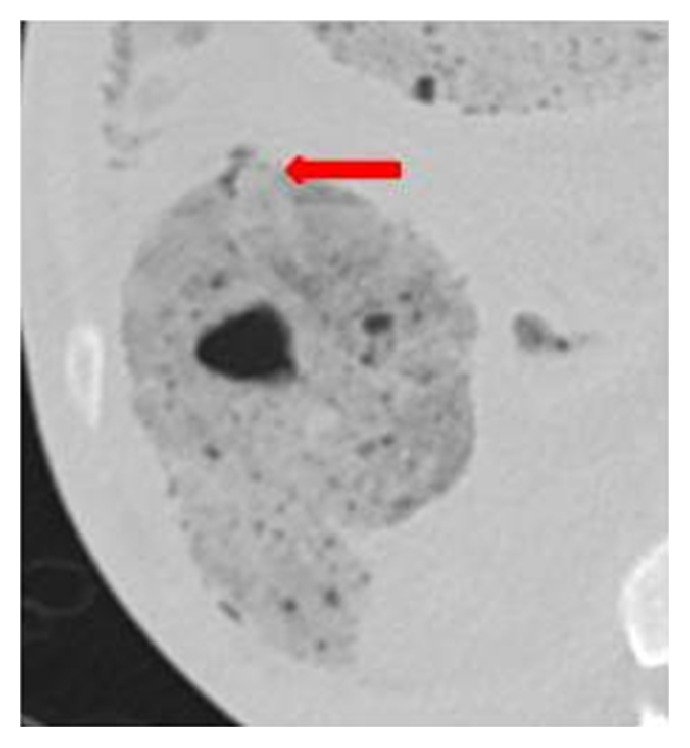
Axial CT image on lung windows (small field of view) confirming the perforation site (arrow).

**Figure 6:**
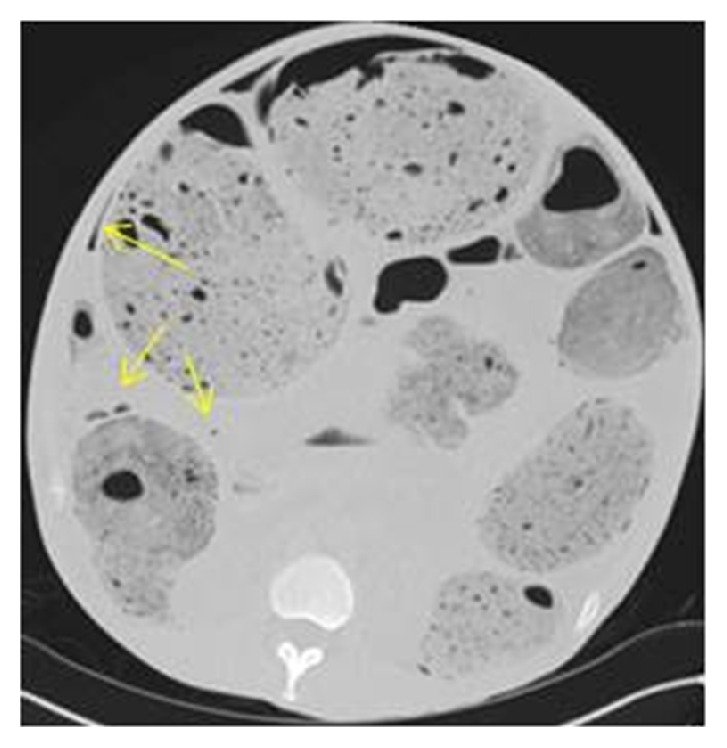
Axial CT images on lung windows confirming small pockets of free gas adjacent to the perforation site.

Blood tests were unremarkable other than an elevated CRP (234). Plain radiographs demonstrated massive faecal loading but nil else (Figures 1 and 2). In view of the patient's clinical stability but diffusely tender abdomen and elevated inflammatory markers, a CT of abdomen and pelvis was performed. The study was noncontrast as the intravenous access had failed. The scan showed significant faecal loading causing massive distension of the colon and anterior ascending colonic perforation (Figures 3 [Fig fig4] [Fig fig5]–6). With the degree of colonic dilatation and lack of intra-abdominal fat, there was a suspicion of free fluid in the abdomen, rendering a stercoral perforation likely. Air was seen in the anterior abdominal wall corresponding well with needle marks on the patient's abdominal surface from injecting heroin.

The patient was consented for laparotomy and proceed and was taken to theatre. At laparotomy, the most obvious abnormality was mega-colon and mega-rectum (30 cm in diameter) with a stercoral perforation of the ascending colon with faecal contamination. Taking into account the patient's physiology and age along with his chronically dilated nonfunctioning colon and rectum, a proctocolectomy was performed with fashioning of an end ileostomy rather than a right hemicolectomy.

The patient was cared for in the High Dependency Unit for 24 hours postoperatively before completing his uneventful recovery on a general surgical ward.

Histological examination of the resected bowel revealed features of thinned bowel wall, ulceration, perforation, and architectural distortion in keeping with distended bowel in megacolon. Ganglion cells were present throughout making the diagnosis of Hirschsprung's disease unlikely. It confirmed the site of perforation within the ascending colon. Microscopically, the muscularis propria was markedly thinned in keeping with the grossly distended appearance of the bowel and the history of megacolon. These features extended throughout the large bowel and terminal ileum. In addition, there was ulceration and focal transmural acute inflammation and necrosis within the ascending colon.

## 3. Discussion

This report describes a case of stercoral perforation of the colon as a result of chronic heroin use. It lends weight to the existing report in the literature advocating the use of preoperative CT imaging in cases of suspected stercoral perforation [5].

The pathophysiology of opioid-induced constipation is well described. Possible aetiologies include increased anal sphincter tone, reduced peristalsis in the small intestine and colon, increased electrolyte and water absorption, and impaired defaecation response [6–11]. Ischaemic pressure necrosis of the colonic wall results from increased intraluminal pressure, leading to stercoral ulcer formation and subsequently colonic perforation [12].

Previous studies have estimated that plain radiographs of stercoral perforation as a consequence of chronic constipation show pneumoperitoneum in only 70% of patients [2], whereas in hollow viscus perforation as a whole, sensitivities in excess of 80% have been reported [13]. One could posit that this figure is lower than expected because megacolon reduces the size of the peritoneal cavity. CT imaging is, therefore, important in detecting small pockets of free air that might not be visible on plain imaging.

## 4. Conclusion

Stercoral perforation of the colon is rare but carries with it significant morbidity and mortality. In this manuscript, we report a case of stercoral perforation due to chronic heroin use. Although this is not the first such case to be reported, it is significant as preoperative CT imaging was influential not only in determining the aetiology of the abdominal distension seen on the plain film, but also in detecting the pneumoperitoneum which was not evident clinically or on plain radiographs.
